# Embed-Search-Align: DNA sequence alignment using Transformer models

**DOI:** 10.1093/bioinformatics/btaf041

**Published:** 2025-02-06

**Authors:** Pavan Holur, K C Enevoldsen, Shreyas Rajesh, Lajoyce Mboning, Thalia Georgiou, Louis-S Bouchard, Matteo Pellegrini, Vwani Roychowdhury

**Affiliations:** Department of Electrical and Computer Engineering, UCLA, Los Angeles, California, 90024, United States; Center for Humanities Computing, Aarhus University, Aarhus, 8000, Denmark; Center for Quantitative Genetics and Genomics, Aarhus University, Aarhus, 8000, Denmark; Department of Electrical and Computer Engineering, UCLA, Los Angeles, California, 90024, United States; Department of Chemistry and Biochemistry, UCLA, Los Angeles, California, 90024, United States; Department of Biochemistry, Biophysics, and Structural Biology (MBIDP), UCLA, Los Angeles, California, 90024, United States; Department of Chemistry and Biochemistry, UCLA, Los Angeles, California, 90024, United States; Department of Molecular, Cell, and Developmental Biology, UCLA, Los Angeles, California, 90024, United States; Department of Electrical and Computer Engineering, UCLA, Los Angeles, California, 90024, United States

## Abstract

**Motivation:**

DNA sequence alignment, an important genomic task, involves assigning short DNA reads to the most probable locations on an extensive reference genome. Conventional methods tackle this challenge in two steps: genome indexing followed by efficient search to locate likely positions for given reads. Building on the success of Large Language Models in encoding text into embeddings, where the distance metric captures semantic similarity, recent efforts have encoded DNA sequences into vectors using Transformers and have shown promising results in tasks involving classification of short DNA sequences. Performance at sequence classification tasks does not, however, guarantee *sequence alignment*, where it is necessary to conduct a genome-wide search to align every read successfully, a *significantly longer-range task by comparison*.

**Results:**

We bridge this gap by developing a “**E**mbed-**S**earch-**A**lign” (ESA) framework, where a novel Reference-Free DNA Embedding (*RDE*) Transformer model generates vector embeddings of reads and fragments of the reference in a shared vector space; read-fragment distance metric is then used as a surrogate for sequence similarity. ESA introduces: (i) Contrastive loss for self-supervised training of DNA sequence representations, facilitating rich reference-free, sequence-level embeddings, and (ii) a DNA vector store to enable search across fragments on a global scale. RDE is 99% accurate when aligning 250-length reads onto a human reference genome of 3 gigabases (single-haploid), rivaling conventional algorithmic sequence alignment methods such as *Bowtie* and *BWA-Mem*. RDE far exceeds the performance of six recent DNA-Transformer model baselines such as *Nucleotide Transformer, Hyena-DNA*, and shows task transfer across chromosomes and species.

**Availability and implementation:**

Please see https://anonymous.4open.science/r/dna2vec-7E4E/readme.md.

## 1. Introduction

Sequence alignment is a central problem in the analysis of sequence data. It is used in various genomic analyses, including variant calling, transcriptomics, and epigenomics. Many DNA sequencers generate short reads that are only a couple of hundred bases long. In order to interpret this data, a typical first step is to align the reads to a genome. Genomes come in many sizes, and commonly studied genomes range from millions of bases (e.g. bacteria) to billions of bases (e.g. mammals, plants) in length. The resulting task of aligning short reads to large-size genomes is computationally challenging—akin to finding a needle in a haystack—and many decades of work have led to optimized approaches that can perform these tasks with great efficiency. Since genomes are sequences where the alphabet consists of only a few symbols ({A,T,G,C}) they can be considered as Limited Alphabet Languages. We ask whether one can design a new paradigm to align reads to genomes by exploiting the Transformer architectures (Vaswani *et al.* 2017) adopted for recent language modeling efforts. The applications of the Transformer model—and, more generally “Large Language Models”—in Bioinformatics applications are still in their infancy, yet hold substantial promise: Transformer models have demonstrated an unprecedented ability to construct powerful numerical representations of sequential data (Baevski *et al.* 2020, Brown *et al.* 2020, Dosovitskiy *et al.* 2021).

We establish a foundation model (Bommasani *et al.* 2021)—Reference-free DNA Embedding (RDE)—tailored for embedding DNA sequences. The model $h_{\Theta }$ (i.e. parameterized by weights Θ) maps any genome subsequence *F* into an embedding $h_{\Theta }(F)\in R^{n}$ (e.g. n=768), such that if *F*_1_ and *F*_2_ are similar subsequences of differing lengths—e.g. *F*_1_ is any noisy subsequence of *F*_2_ or *F*_1_ and *F*_2_ have significant overlaps—then the $d(h_{\Theta }(F_{1}),h_{\Theta }(F_{2}))$ is small; where d(·) is a distance metric in the embedding space, such as the cosine distance between two vectors. Another requirement of such RDE models is that they should generate embeddings that are reference free, i.e. once trained, it creates semantically aware embedding (i.e. sequences that are close in edit distance are mapped close to each other) of any given genome subsequences *irrespective of the reference genome from which it is sampled*. Given such a model, alignment of reads can be achieved by a near-neighbor search in the embedding space: only the fragments of the genome with embeddings nearest to the embedding of the given read need to be considered. Thus, a global search in a giga-bases long sequence is reduced to a local search in a vector space.

### 1.1 Transformer models: written language to DNA sequence alignment

DNA sequences share remarkable similarities with written language, offering a compelling avenue for the application of Transformer models. Like written language, these are sequences generated by a small alphabet of nucleotides {A,T,G,C}. Traditional DNA modeling efforts have already accommodated mature encoding and hashing techniques initially developed for written language—such as Suffix trees/arrays and Huffman coding (Huffman 1952, Manber and Myers 1993)—to successfully parse and compress DNA sequences. Alignment methods such as MinHash and MashMap (Kille *et al.* 2023) have incorporated *Locality Sensitive* Hashing (LSH) (Marçais *et al.* 2019) to construct fast, approximate representations of DNA sequences that facilitate the rapid matching of millions of reads onto the reference genome.

Within the last few years, several Transformer-based models have been developed for DNA sequence analysis. Notably, DNABERT-2 (Ji *et al.* 2021, Zhou *et al.* 2023), Nucleotide Transformer (Dalla-Torre *et al.* 2024), GenSLM (Zvyagin *et al.* 2023), HyenaDNA (Nguyen *et al.* 2023), and GENA-LM (Fishman *et al.* 2025) have been designed to discern relationships between short genetic fragments and their functions. Specifically, Nucleotide Transformer representations have shown utility in classifying key genomic features such as enhancer regions and promoter sequences. Similarly, GENA-LM has proven effective in identifying enhancers and Poly-adenylation sites in Drosophila. In parallel, DNABERT-2 representations have also been found to cluster in the representation space according to certain types of genetic function.

These models, trained on classification tasks, generate sequence embeddings such that their pairwise distances correspond to class separation. As a result, pairs of sequences with very large edit distances between them are mapped to numerical representations that are close by. However, in tasks such as Sequence Alignment the objective is quite different: *the pairwise representation distance has to closely match the sequence edit distance*. A natural question arises: *Can these Transformer architectures be readily applied to the task of Sequence Alignment?* We delineate the associated challenges as follows:

**[L1] Two-stage training:** DNA-based Transformer models typically undergo pretraining via a *Next Token/Masked Token Prediction* framework, a method originally developed for natural language tasks. To form sequence-level representations, these models often use pooling techniques that aggregate token-level features into a single feature vector. This approach, however, is sometimes critiqued for yielding suboptimal aggregate features (Reimers and Gurevych 2019).
**[L2] Computation cost:** The computational requirements for Transformer models grow quadratically with the length of the input sequence. This is particularly challenging for sequence alignment tasks that necessitate scanning entire genomic reference sequences.

**Figure 1.**
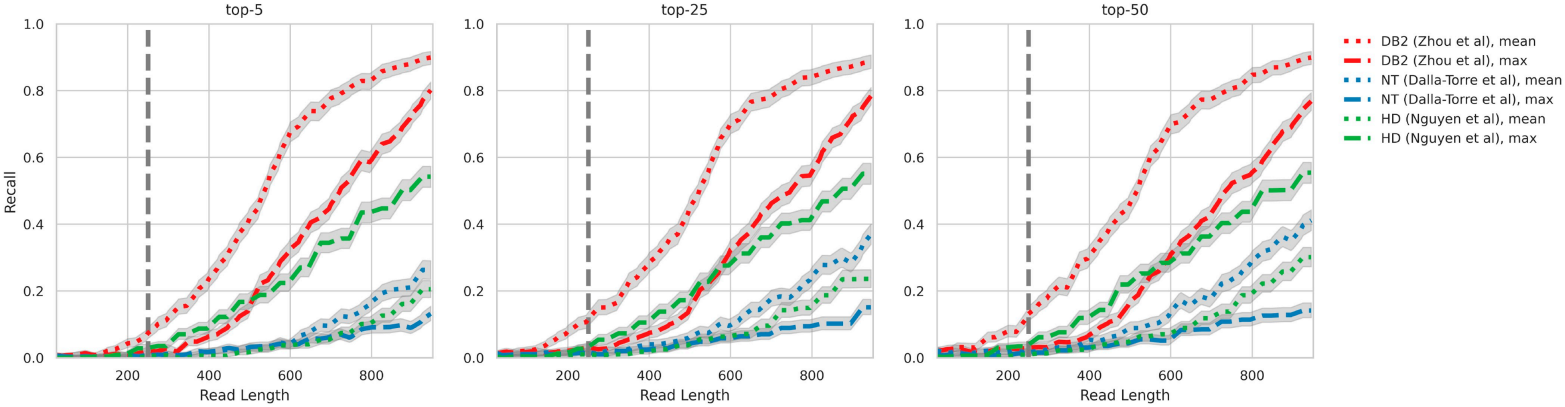
Alignment recall of transformer-DNA baselines by read length: existing transformer-DNA models were adapted for sequence alignment using mean-/max-pooling. Their performance, measured by recall (top-*K*) over 40*K* reads of varying lengths across the human genome (3 gb—single-haploid), is shown. Trendlines represent each baseline, with error bars [Clopper–Pearson Interval (Clopper and Pearson 1934)) @ 95%] in grey. The vertical line at *x *=* *250 marks a typical read length. Overall, these baselines show suboptimal performance. For more details, see Section 3.

Figure 1 shows the sequence alignment performance (recall) of several Transformer-DNA models. The testing protocols are elaborated in Section 3. Notably, these models exhibit subpar recall performance when aligning typical read lengths of 250.

## 2 Our contributions

In this paper, we argue that both limitations **L1**, **L2** of Transformer-DNA models can be mitigated by formulating sequence alignment as a vector search-and-retrieval task. Our approach is 2-fold: (A) We introduce a sequence encoder *Reference-Free DNA Embedding* (RDE), trained through self-supervision, to embed DNA subsequences to vectors. (B) Next, we formulate a framework, *Embed-Search-Align* (ESA), that maps reads to reference genome sequence. We leverage a specialized data structure, termed a *DNA vector store*, that enables efficient storage and search in an embedding space (Codebase available here: https://anonymous.4open.science/r/dna2vec-7E4E/): the entire reference genome is sharded into equal-length overlapping fragments whose embeddings are then uploaded to the vector store. For each read, top-K closest fragments can then be searched efficiently in time that scales only logarithmically in the number of fragments (Malkov and Yashunin 2018). These strategies have been explored in NLP: (A) Sequence-to-embedding training using contrastive loss has shown improved performance—over explicit pooling methods—at abstractive semantic tasks such as evidence retrieval (Lewis *et al.* 2020) and semantic text similarity (Chen *et al.* 2020, Gao *et al.* 2021). (B) Specialized data structures, such as “vector stores” or “vector databases” like FAISS (Johnson *et al.* 2021) and *Pinecone*, use advanced indexing and retrieval algorithms for scalable numerical representation search.

### 2.1 Task definition

The simplest sequence alignment task applies to single-end (A DNA fragment is ligated to an adapter and then sequenced from one end only.) reads, where a sequencer generates a read of length *Q*
 

(1) $$r:=({\tilde{b}}_{1},{\tilde{b}}_{2},\ldots ,{\tilde{b}}_{Q}),$$

where ${\tilde{b}}_{i}\in \{A,T,G,C\}$. In practice, these reads come from individual genomes that do not necessarily match the reference and may contain mutations due to base insertions, deletions, and substitutions. Thus, it is assumed that this read is a noisy substring taken from a reference genome sequence $R:=(b_{1},b_{2},\ldots ,b_{N}),b_{i}\in \{A,T,G,C\}$ and N≫Q; e.g. for the single-haploid human genome (Nurk *et al* 2022), N≈3 gigabases (gb), and Q≈250 though reads of much longer lengths are becoming increasingly affordable and accurate (Zook *et al.* 2016). The alignment task is to find a substring in R
 

(2) $$\tilde{r}:=(b_{q},b_{q+1},\ldots ,b_{q+Q}),1\leq q\leq N-Q,$$

such that the edit distance $d(r,\tilde{r})$—computed using the Smith-Waterman (SW) algorithm—is minimized. The primary objective is to identify the most probable location, *q*, of this read within the reference genome.

We formulate the problem of Sequence Alignment as minimizing a sequence alignment function, SA, applied to a read *r* and a reference sequence R as

(3) $$v^{*}=\underset{q}{\min}\mathrm{SA}(r,R)$$

where q∈{1,2,3,…} is a candidate reference starting position and $v^{*}$ is the optimal alignment score. Lower scores indicate better alignments. This optimization exhibits the following property:

#### 2.1.1 Sharding for sequence alignment

For a read segment *r* of length *Q* and reference R of length *N*, the complexity of SA(r,R) scales as O(NQ), which is expensive.

Ideally, we would like to use R to get a set of fragments $\left\{F_{1},F_{2},\ldots ,F_{K}\right\}$ that are a subset of the original reference. Then:

(4) $$v^{*}\approx \underset{F_{j}\in \{F_{1},F_{2},\ldots ,F_{K}\}}{\min}\mathrm{SA}\left(r,F_{j}\right).$$

Here, each $F_{j}$ is a fragment of R (i.e. $F_{j}\subset R$), and *K* is the number of these sub-tasks. This approximation is effective under the conditions:

1. Fragment $F_{j}$ lengths are on the order of the read length (*Q*), and not the length of the longer reference R;
2. Since a read can originate from any position along the reference, there must be sufficient fragments $F_{i}$ to cover R, i.e. $\cup F_{j}=R$. From within this set of fragments, we hope to retrieve a subset of fragments containing the optimal fragment for the read;
3. While retrieving a subset of fragments of size *K*, we require *K* to be significantly smaller than $\frac{N}{Q}$. If $\frac{N}{Q}$, then this amounts to scanning the whole reference.

**Figure 2.**
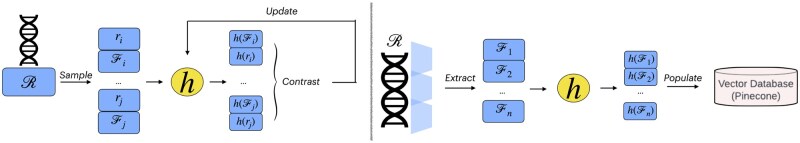
System overview [A]—training encoder and populating vector store: reference genome fragments $F_{i}$ and within them, randomly sampled pure reads *r_i_* (positive pairs) are numerically represented via shared encoder *h*. Encoder training follows a contrastive approach as per (6). After training, the genome is segmented into overlapping fragments, encoded, and uploaded into the vector store.

**Figure 3.**
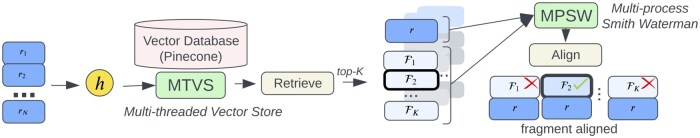
System overview [B]—inference on a new read: a read, as per (1) and generated by ART (Huang *et al.* 2012), is encoded by *h*. This is then compared to reference fragment representations in the vector database. The nearest-*K* fragments in the embedding space are retrieved for each read, and the optimal alignment is determined using (7).

Conditions (1) and (2) imply that fragments should be short and numerous enough to cover the reference genome. Condition (3) restricts the number of retrieved reference fragments per read—that we deem to be most likely to contain *r*—to a small value *K*. Analogous methods have shown efficacy in text-based Search-and-Retrieval tasks (Dai *et al.* 2022, Peng *et al.* 2023) on Open-Domain Question-Answering, Ranking among other tasks. Subsequent sections describe a parallel framework for retrieving reference fragments given a read. The pipeline is shown in Figs 2 and 3.

### 2.2 RDE: designing effective sequence representations

An optimal sequence encoder model *h* is such that the corresponding embeddings of any read *r* and reference fragment F—h(r),h(F) respectively—obey the following constraints over a pre-determined distance metric *d*:

(5) $$d\{h(r_{j}),h(F_{i})\}\geq d\{h(r_{j}),h(F_{j})\},\;i\neq j$$

Subscript *j* serves to indicate that the read *r_j_* is *correctly aligned to* a fragment reference, $F_{j}$, (*that we call a positive {read, fragment} pair*). Given another arbitrary fragment $F_{i}$ to which *r_j_* has a poor alignment, $\left\{r_{j},F_{i}\right\}$ is a negative {read, fragment} pair. Observe that these inequalities constitute the only requirements for the encoder. As long as the *neighborhood* of *r_j_* in the representation space *contains* the representation for $F_{j}$, it will be recovered in the nearest neighbors (top-*K* set) and alignment will succeed. Equality is observed when *r_j_* is a repeat sequence matched equally well to more than one fragment. This motivates using self-supervision (Hadsell *et al.* 2006, Chen *et al.* 2020, Gao *et al.* 2021) where we are only concerned about the relative distances between positive and negative (read, reference fragment) pairs.

### 2.3 RDE: self-supervision and contrastive loss

A popular choice for sequence learning using self-supervision involves a contrastive loss setup described by Chen *et al.* (2020) and Gao *et al.* (2021): i.e. for a read *r* aligned to reference fragment $F_{j}$, the loss *l_r_* simultaneously minimizes the distance of *h*(*r*) to $h(F_{j})$ and maximizes the distance to a batch of random fragments of size *B—*1:

(6) $$l_{r}=-\text{log}\;\frac{e^{-d(h(r),h(F_{j}))/\tau }}{e^{-d(h(r),h(F_{j}))/\tau }+\sum_{i=1}^{B-1}e^{-d(h(r),h(F_{i}))/\tau }}.$$

Here *τ* is a tuneable temperature parameter. To stabilize the training procedure and reach a nontrivial solution, the encoder applies different dropout masks to the reads and fragments similar to the method described in prior work (Chen *et al.* 2020). Similar setups have been shown to work in written language applications, most notably in Sentence Transformers (Reimers and Gurevych 2019, Gao *et al.* 2021, Muennighoff *et al.* 2023), which continue to be a strong benchmark for several downstream tasks requiring pre-trained sequence embeddings.

### 2.4 RDE: encoder implementation

RDE uses a Transformer-encoder (Vaswani *et al.* 2017, Devlin *et al.* 2019), comprising 12 heads and 6-layers of encoder blocks. The size of the vocabulary is 10, 000. Batch size *B* is set to 16 with gradient accumulation across 16 steps. Generated numerical representations for each read (and fragment) are projected into a high-dimensional vector space, $h(r)\in R^{1020}$. The learning rate is annealed using one-cycle cosine annealing (Smith and Topin 2019), dropout is set to 0.1, and τ=0.05. During training, the reference fragment $\left|F_{i}|∼U([800,2000]\right)$ and read $\left|r_{i}|∼U([150,500]\right)$ have variable lengths sampled from a uniform distribution. Here, |x| denotes the length of *x*. To improve model performance on noisy reads, 1−5% of bases are replaced with another random base in 40% of the reads in a batch. Shorter sequences were padded to equal the length of the longest sequence in a batch. The similarity measure used is *Cosine Similarity*.

### 2.5 ESA: search and retrieval

An outline of the search and retrieval process is presented in Fig. 3. Every read is encoded using the trained model and matched to reference fragments in the vector database. The top-*K* retrieved fragments per read are then aligned using a SW alignment library to find the optimal alignment. The following sections describe the indexing and retrieval part in more detail.

#### 2.5.1 Indexing

For a given reference genome R, we construct a minimal set of reference fragments $F:=\{F_{1},F_{2},\ldots \}$ to span R. Note that the fragments overlap at least a read length; i.e. $|F_{i}\cap F_{i+1}|\geq Q$ to guarantee that every read is fully contained within some fragment in the set. In our experiments with external read generators (Huang *et al.* 2012), $Q_{\max}=250,|F_{i}|=1250$. Each reference fragment is encoded using the trained *RDE* model, and the resulting sequence embeddings ($\in R^{1020}$)—3M vectors for a reference of 3B nucleotides—are inserted into a Pinecone (https://www.pinecone.io) database. Once populated with all the fragments, we are ready to perform the alignment.

#### 2.5.2 Retrieval

Given a read *r*, we project its corresponding *RDE* representation into the vector store and retrieve the approximate nearest-*K* set of reference fragment vectors and the corresponding fragment metadata $\left\{F_{1},F_{2},\ldots ,F_{K}\right\}$.

#### 2.5.3 Diversity priors

While the top-*K* retrieved fragments can be drawn from across the entire vector store (genome), contemporary recommendation systems that use the top-*K* retrieval setup *rank and re-rank* top search results (*Slate Optimization—*see Zhu *et al.* 2007) to ensure rich and diverse recommendations. Similarly, we apply a uniform prior wherein every retrieval step selects the top-*K per* chromosome.

#### 2.5.4 Fine-alignment

A standard SW score library (Cock *et al.* 2009) is used to solve (4), which can be executed concurrently across the *K*-reference fragments. Let the optimal fragment be $F^{*}$. The metadata for each vector includes (a) the raw $F^{*}$ sequence; (b) the start position of $F^{*}$ within the reference $R,\;q_{F^{*}|R}$. Upon retrieval of a fragment and fine-alignment to find the fragment-level start index, $q_{|F^{*}}$, the global reference start position is obtained as:

(7) $$q^{*}=q_{|F^{*}}+q_{F^{*}|R}.$$

## 3 Transformer-DNA baselines

This section outlines the setup for evaluating Transformer-DNA baselines, with their recall performance depicted in Fig. 1. We selected three architectures modeling nucleotide sequences: [NT] *NucleotideTransformer* ($\in R^{1280}$) (Dalla-Torre *et al.* 2024), [DB2] *DNABERT-2* ($\in R^{768}$) (Ji *et al.* 2021), and [HD] *HyenaDNA* ($\in R^{256}$) (Nguyen *et al.* 2023). Each model uses mean- and max-pooling of token representations for sequence encoding (2 × 3 = 6 baselines total). Independent vector stores for each baseline encode fragments from the entire 3gb genome. We sampled 40K pure reads (reads without noise in comparison to the reference) of varying lengths (Q∼U([25,1000])) and assessed the average recall for top-5, top-25, and top-50 fragments, as shown in Fig. 1. Overall, while baseline performance is modest, mean-pooling generally outperforms max-pooling, with DB2 (mean-pooled) and HD (max-pooled) as the most effective. These two baselines will be contrasted with RDE on ESA in Table 1.

**Table 1.** Performance of RDE with respect to baselines: the performance of RDE on ESA is compared to the DNA-BERT 2 and Hyena-DNA Transformer-based baseline models (with no additional finetuning)—the top performing baselines from Fig. 1.^a^

Model	Settings	ART (MiSeqv3)				PacBio CCS ($\mu (v^{*}),\sigma (v^{*})$)
	$I,D=0.01,Q=250,\;d_{SW}=2\%$	Search	[10,30]	[30,60]	[60,90]	Chr. 2, pbmm2 $d_{SW}=5\%$
Transformer architectures	HD (max)	K = 50	13.20 ± 1.81	17.01 ± 2.01	18.03 ± 2.05	
	DB2 (mean)		30.21 ± 2.44	39.19 ± 2.58	39.20 ± 2.58	
	**RDE (ours)**		98.40 ± 0.71	98.64 ± 0.67	98.60 ± 0.67	97.5 ± 0.82
		K = 75	**98.80 ± 0.62**	**99.28 ± 0.52**	**98.88 ± 0.60**	**97.5 ± 0.82 (−498.0, 3.78)**
Bowtie-2 (Classical)			99.80 ± 0.19			99.50 ±0.43 (−497.9, 3.78)
*diff.*			<1%			<2%

a In addition, RDE is also compared with Bowtie-2 (Langmead *et al.* 2019), a classical aligner. Comparisons are conducted across varying qualities (*Phred score*) of reads generated by ART (Huang *et al.* 2012). Additionally, an external PacBio CCS dataset of (*Q *=* *250 length-) reads from Chr. 2 of the Ashkenazim Trio—Son sample [as determined by pbmm2 (Li 2018)] are aligned using both Bowtie-2 and RDE. All baseline models utilize a dedicated vector store. For details on $I,D,Q_{PH},K,d_{SW}$, refer to *Recall/Simulator Configurations*. Across models and ART-generated datasets, the performance of RDE (in bold) supersedes other Transformer-based models and is comparable to Bowtie-2 to within 1%. With respect to reads from PacBio CCS, RDE performs with 2% of Bowtie-2 after controlling for the intrinsic quality of retrieved fragments according to the SW score. Mean (*μ*) and standard deviation (*σ*) across the top SW scores ($v^{*}$) of reads successfully aligned in the case of RDE and Bowtie-2 models are reported.

**Figure 4.**
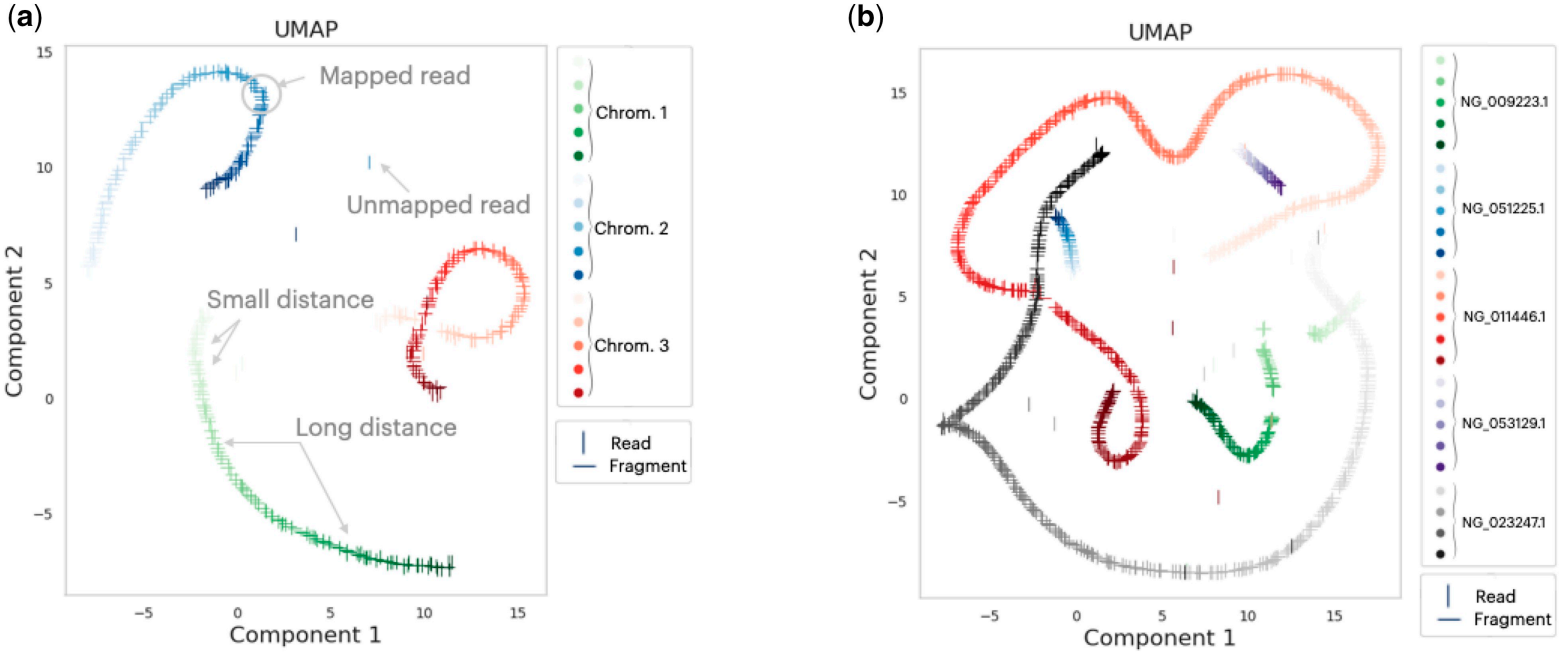
Illustrating RDE’s preservation of sequence locality in embedding space: the reference genome R is divided into fragments $F_{i}$, each represented by an embedding $h(F_{i})$. For effective sequence alignment, specific structures are expected in the embedding space: (i) overlapping fragments $F_{i}$ and $F_{j}$ should have proximate embeddings; (ii) consecutive fragments forming a long sequence should correspond to a distinct manifold in the embedding space. Subfigures (a) and (b) display this emergent geometry, as visualized using embeddings produced by RDE. In both subfigures, the fragment and read representations are jointly visualized in a 2D low-dimensional space constructed by applying UMAP (McInnes *et al.* 2018) to the original embeddings. Vertical marker lines (|) refer to fragments, “–” markers correspond to reads. We observe that the consecutive fragments belonging to the same nucleotide (subfigure a) or gene (subfigure b) sequence constitute an order-preserving 1D manifold. Additionally, *almost all of the reads* are close to their corresponding fragments, making this space viable for the task of Sequence Alignment: by searching for fragments in the neighborhood of an external read represented in the embedding space, one is likely to retrieve a fragment most responsible for the read.

RDE convergence plots are presented in the Supplementary Section S1. Model checkpoints are available in OSF (Holur *et al.* 2023). In Fig. 4, representations of short 1000-length sequences sampled from sequential (in-order) and gene-specific locations in the reference are visualized in a reduced 2D-UMAP (McInnes *et al.* 2018). The representation space demonstrates desired properties suitable for successfully performing alignment: (i) sequences sampled in order form a trajectory in the representation space: the loss function described in (6) encourages a pair of sequences *close* to one another to have a short distance between them in the representation space, and pairs further apart to have a larger distance. (ii) Representations of sequences drawn from specific gene locations—despite not being close to one another—show gene-centric clustering: the RDE representation space partially acquires function-level separation as a byproduct of imposing *local* alignment constraints. The codebase is *linked*.

## 4 Sequence alignment of ART-simulated reads

The results from Section 3 demonstrate that even for pure reads, baseline models do not generate adequate representations to perform sequence alignment. In this section, RDE and the two best baselines—*DB2, mean*, and *HD, max*—are evaluated on ESA using reads generated from an external read simulator (ART)—see Huang *et al.* (2012). ART has served as a reliable benchmark for evaluating other contemporary alignment tools and provides controls to model mutations and variations common in reads generated by Illumina machines.

### 4.1 Simulator configurations

The different simulation configuration options and settings are listed: (i) *Phred quality score Q_PH_* in one of three {[10,30],[30,60],[60,90]} ranges: the likelihood of errors in base-calls of a generated read; (ii) *Insertion rate* $I\in \{0,10^{-2}\}$: the likelihood of adding a base to a random location in a read; (iii) *Deletion rate* $D\in \{0,10^{-2}\}$: the likelihood of deleting a base in the read; (Others): *Simulator system*: MSv3 [MiSeq]; *Read length*: 250.

### 4.2 Recall configurations

Once the top-K fragments have been retrieved, the first step is to solve (4), and for this, we need to compute the SW score. For all presented results, the settings are: match_score = −2, mismatch_penalty = +1, open_gap_penalty = +0.5, continue_gap_penalty = +0.1. After alignment, we get $q^{*}$—see (7)—as the estimated location of a read in the genome. Let ${\hat{q}}^{*}$ be its true location. If $q^{*}={\hat{q}}^{*}$, it is a perfect match and the recall is successful. In cases where there is a mutation in the first or last position in a read, the fine-alignment will return $q_{F^{*}}^{*}$ offset by at most two locations, resulting in ${\hat{q}}^{*}=q^{*}\pm 2$. Hence, the condition for an exact location match: $|q^{*}-{\hat{q}}^{*}|\leq 2$.

### 4.3 Distance bound, $d_{SW}\in \{1\%,2\%,5\%\}$

It is well known that short fragments frequently repeat in the genome and $q^{*}$ can correspond to the position of the read in a different location than from where it was sampled (Simkin and Roychowdhury 2011, Li and Freudenberg 2014). In this case, $q^{*}\neq {\hat{q}}^{*}$, but the SW score is the minimum possible. Moreover, when reads have mutations, the reference sequence corresponding to the read is no longer a perfect match. We define a bound *d*_SW_ for classifying whether a (read, retrieved fragment) pair is a successful alignment based on the SW score between the pair ($v^{*}$—see Equation 4) and the optimal SW score as a particular read length *Q*:

$$d_{SW}=\frac{v^{*}-mQ}{(n-m)Q},$$

where m(=−2) is the match score and n(=+1) is the mismatch penalty, hyperparameters in the computation of the SW score. We consider an alignment (with *Q *=* *250) to be successful if the resulting SW score between the read and the top returned fragment is within *d*_SW_ of the optimal for that read length. A $d_{SW}=2\%$ (*default*) is equivalent to a mismatch of around 4 bases in a read of length 250.

### 4.4 Performance

Table 1 reports the performance of RDE on ESA across several read generation configurations—we choose from one of three ranges of Phred score *Q_PH_*, {[10,30],[30,60],[60,90]}—$I,D=0.01,\;d_{SW}=2\%$ (see Section 4), in addition to a direct comparison to DB2, mean, and HD, max baselines (*without any further finetuning*), the best-performing baselines on pure reads identified in Section 3. In addition, we also compare RDE to Bowtie-2 (Langmead *et al.* 2019), a conventional algorithmic aligner.

Additionally, models are also evaluated on an external set of reads from PacBio CCS generated on the Ashkenazim Trio (Son) (retrieved from the Genome in a Bottle resource (GIAB) (Zook *et al.* 2016)). For (ii), the raw reads are 10 kilobases long and for evaluation we consider random subset of 5000 reads associated to Chr. 2. Reads input into the aligner are cropped randomly to a length of 250 to satisfy the computational requirements of RDE ($|F_{i}\cap F_{i+1}|=250$).

We observe that: (i) RDE shows strong recall performance of >99% across a variety of read generation and recall configurations; (ii) On cleaner reads, RDE and Bowtie-2 are within <1% of each other in recall while controlling for the quality of the retrieved alignments (as determined by the SW score). The results indicate that this constitutes a new state-of-the-art model for Transformer-based sequence alignment.

### 4.5 Sweeping Top-K and the SW distance bound

In Table 2, we report the performance of RDE across distance bounds $d_{SW}\in \{1\%,2\%,5\%\}$ and a number of recalled fragment settings K∈{25,50,75}. Distance bound $d_{SW}<5\%$ is equivalent to an acceptable mismatch of at most ∼8 bases between the read (length *Q *=* *250) and recalled reference fragments. Recall rates are comparable with different *d*_SW_ values suggesting that when a read is successfully aligned, it is usually aligned to an objectively best match. Decreasing the top-*K* per chromosome from 50→25 does not substantially worsen performance (<1%), indicating that the optimal retrievals are usually the closest in the embedding space. Several additional experiments are presented in the Supplementary Material.

**Table 2.** Sequence alignment recall of RDE sweeping top-K and *d_SW_*: the various parameters are described in Section 4.^a^

*I*	*D*	$Q_{PH}\in [30,60]$			$Q_{PH}\in [60,90]$		
		$d_{SW}<1\%$	$d_{SW}<2\%$	$d_{SW}<5\%$	$d_{SW}<1\%$	$d_{SW}<2\%$	$d_{SW}<5\%$
Top-25/chromosome							
0.0	0.0	97 ± 0.94	98.12 ± 0.76	98.56 ± 0.69	97.24 ± 0.91	97.96 ± 0.0.80	98.6 ± 0.69
	0.01	96.88 ± 0.97	98 ± 0.78	98.36 ± 0.73	97.24 ± 0.91	97.96 ± 0.80	98.76 ± 0.64
0.01	0.0	97.08 ± 0.93	98.04 ± 0.78	98.56 ± 0.69	97 ± 0.94	98.24 ± 0.75	98.92 ± 0.59
	0.01	97.16 ± 0.80	97.96 ± 0.56	99.2 ± 0.52	97.6 ± 0.85	98.12 ± 0.76	98.76 ± 0.62
Top-50/chromosome							
0.0	0.0	97.52 ± 0.86	99.04 ± 0.57	99.08 ± 0.57	98.2 ± 0.75	98.88 ± 0.59	99.16 ± 0.52
	0.01	97.48 ± 0.87	99.16 ± 0.52	99.08 ± 0.55	97.8 ± 0.82	98.76 ± 0.62	99.04 ± 0.57
0.01	0.0	98.24 ± 0.75	98.64 ± 0.67	99.12 ± 0.55	98.28 ± 0.75	98.92 ± 0.59	99.28 ± 0.49
	0.01	97.72 ± 0.83	98.64 ± 0.67	99.36 ± 0.46	97.84 ± 0.92	98.6 ± 0.66	99.0 ± 0.57
Top-75/chromosome							
0.0	0.0	98.04 ± 0.78	99.12 ± 0.55	99.0 ± 0.57	98.4 ± 0.71	99.12 ± 0.55	98.4 ± 0.71
	0.01	98.4 ± 0.71	99.04 ± 0.56	99.4 ± 0.46	98.44 ± 0.71	98.84 ± 0.62	99.24 ± 0.52
0.01	0.0	98.64 ± 0.67	99.0 ± 0.57	99.2 ± 0.52	98.0 ± 0.78	98.72 ± 0.64	99.48 ± 0.43
	0.01	98.68 ± 0.64	99.28 ± 0.49	99.4 ± 0.46	98.48 ± 0.69	98.88 ± 0.59	99.4 ± 0.46

a RDE presents a recall of >99% across several read configurations rivaling contemporary algorithmic models such as Bowtie. As expected, performance improves with larger search radius (top-*K*), higher quality reads (*Q_PH_*), and large distance bound *d_SW_*.

## 5 Limitations and future work

While our first-generation reference-free DNA embedding (RDE) models provide alignment performance that almost matches the performance of traditional aligners (refined over decades), there is room for considerable improvement. First, our RDE models have been trained using samples that span only around 2% of the human genome as shown in the Supplementary Section S1 of the Supplementary Material. One needs to explore if the embedding properties of our models can be further improved by diversifying and augmenting training data and optimizing over the model parameters. The validation of RDE models can be performed only through various genomic tasks. In terms of the task of alignment, we plan to explore the following: (i) the current off-the-shelf implementation of our ESA algorithm has a speed of around 10K reads per minute. The existing aligners such as Bowtie are faster, and can achieve close to 1M reads per minute; additional speedups can be achieved by trading off alignment accuracy (Darby *et al.* 2020). As detailed in Supplementary Section S2, we are considering various optimization strategies, including model compilation, to speed up inference and enhanced parallelization in vector store searches and fragment-read alignment. We believe ESA can be made much faster; (ii) optimization of the training of the RDE models to improve performance for shorter reads. As shown in see Table 2, Page 3 of SM the existing model performs well on longer reads; we want to develop RDE models that specialize to aligning shorter reads; and (iii) quantifying how embeddings change as subsequences are edited; this will help in better alignment accuracy. It will also help in correlating the geometry of the embedding space to the structure of DNA sequences.

## 6 Concluding remarks

We have introduced a novel Reference-Free DNA Embedding (RDE) framework: it creates an embedding of any given DNA subsequence irrespective of the reference genome from which it is sampled. Such models capture similarity among genome subsequences in a *purely data-driven manner* (without explicitly computing edit-distance measures) and embeds DNA sequences of differing lengths into the same embedding space to facilitate search and assembly. Once such RDE models are constructed their expressive power and limitations can be assessed by performing multiple challenging genomic tasks in a flexible manner across different species.

As a first validation and application of RDE, we developed an Embed-Align-Search (ESA) framework where alignment of reads to a reference genome is achieved by a near-neighbor search in the embedding space: only the fragments of reference genome with embeddings nearest to the embedding of the given read need to be considered. Thus, a global search in a giga-bases long reference genome sequence is reduced to a local search in a vector space. Detailed experiments showed that the performance of even this first-generation RDE methodology is comparable to that of traditional aligners, which have been refined over decades. Future application of RDE to the alignment task might lie in aligning reads to pan-genomes (Liao *et al.* 2023), where the diversity in the genomic content—modeled as allelic paths in a reference genome graph—can be exhaustively mapped onto the described embedding space (as in Fig. 4). Conveniently, the proposed nearest-neighbor search would align a given read to fragments; the only difference being that multiple fragments can belong to the same reference location but different reference paths. The ESA methodology would not need to be modified beyond this.

An important question we want to answer going forward is: How reference-free is our RDE model? To benchmark the generalizability of our RDE model we have performed several cross-species alignment tasks as summarized in Supplementary Tables S3 and S4 in the Supplementary Material. In particular, we have used an RDE trained using the human genome to align reads to reference genomes belonging to a wide range of species. The results are encouraging and the design of universal RDEs—that would generate effective embeddings of subsequences sampled from the genome of every species—is another direction of future research.

One can also harness the RDE space to address the complementary task of *de novo Genome Assembly*, where given a set of reads one assembles a genome by stitching together the reads, without using any reference. Genome assembly is considered to be a much more challenging task than alignment. In Supplementary Section S6, we provide initial proofpoints of a genome assembly framework, under reasonable simplifying assumptions about read lengths and overlaps among the reads. We show how the RDE model—trained on exemplars from the human genome—can effectively embed simulated reads from a very different species, Thermus Aquaticus. We find that the read embeddings form a low-dimensional manifold, which can be efficiently searched locally to obtain high-fidelity genome assembly from the simulated reads. Our future work on genome assembly will focus on improving RDE models to obtain better embeddings, and also on developing local-search based algorithms that can accurately map low-dimensional manifolds (comprised by the embeddings of reads) into linear DNA sequences.

## Supplementary Material

btaf041_Supplementary_Data
