# Supplementary material for: Embed-Search-Align: DNA sequence alignment using Transformer models
Source: Bioinformatics. 2025 Feb 6;41(3):btaf041. doi: 10.1093/bioinformatics/btaf041 (PMC11919449; doi:10.1093/bioinformatics/btaf041)
Supplement: btaf041_Supplementary_Data [file btaf041_supplementary_data.zip › 81f1d_ESA_Final_Bioinformatics_Main (9).pdf]

Bioinformatics, 2023, pp. 1–6

doi: DOI HERE
Advance Access Publication Date: Day Month Year
Suppporting File

SUPPPORTING FILE

Supplementary Information: ESA

Pavan Holur,[1,†] K. C. Enevoldsen,[2,3,†] Shreyas Rajesh,[1] Lajoyce Mboning,[4] Thalia Georgiou,[5] Louis-S. Bouchard,[4] Matteo Pellegrini[6] and Vwani Roychowdhury[1,†]

[1]Department of Electrical and Computer Engineering, UCLA, [2]Center for Humanities Computing, Aarhus University, [3]Center for Quantitative Genetics and Genomics, Aarhus University, [4]Department of Chemistry and Biochemistry, UCLA, [5]Department of Biochemistry, Biophysics, and Structural Biology (MBIDP), UCLA and [6]Molecular, Cell, and Developmental Biology, UCLA
[*]Corresponding author. vwani@g.ucla.edu
FOR PUBLISHER ONLY Received on Date Month Year; revised on Date Month Year; accepted on Date Month Year

Abstract

Motivation: DNA sequence alignment, an important genomic task, involves assigning short DNA reads to the most probable locations on an extensive reference genome. Conventional methods tackle this challenge in two steps: genome indexing followed by efficient search to locate likely positions for given reads. Building on the success of Large Language Models (LLM) in encoding text into embeddings, where the distance metric captures semantic similarity, recent efforts have encoded DNA sequences into vectors using Transformers and have shown promising results in tasks involving classification of short DNA sequences. Performance at sequence classification tasks does not, however, guarantee *sequence alignment*, where it is necessary to conduct a genome-wide search to align every read successfully, a *significantly longer-range task by comparison. Results:* We bridge this gap by developing a "**E**mbed-**S**earch-**A**lign" (ESA) framework, where a novel Reference-Free DNA Embedding (*RDE*) Transformer model generates vector embeddings of reads and fragments of the reference in a shared vector space; read-fragment distance metric is then used as a surrogate for sequence similarity. ESA introduces: (1) Contrastive loss for self-supervised training of DNA sequence representations, facilitating rich reference-free, sequence-level embeddings, and (2) a DNA vector store to enable search across fragments on a global scale. RDE is 99% accurate when aligning 250-length reads onto a human reference genome of 3 gigabases (single-haploid), rivaling conventional algorithmic sequence alignment methods such as *Bowtie* and *BWA-Mem*. RDE far exceeds the performance of 6 recent DNA-Transformer model baselines such as *Nucleotide Transformer, Hyena-DNA*, and shows task transfer across chromosomes and species. *Availability and Information:* Please see `https://anonymous.4open.science/r/dna2vec-7E4E/readme.md`. *Contact:* Pavan Holur (`pholur@g.ucla.edu`).

Key words: Transformers, DNA Sequence Alignment, Large Language Models, Vector Stores

Appendix

A. Training Convergence and Data Usage

Fig. 1 plot the convergence of the RDE encoder model discussed in the main text. We see convergence after ∼2k steps.

With 2000 steps and a batch size of 16, we have a total of $2000 \times 16 = 32,000$ training fragments. The maximum size of a fragment is 2000 base pairs. Therefore, the total number of base pairs seen during training is approximately $32,000 \times 2000 = 64000000$ (64 million) base pairs.The human genome consists of around 3 billion base pairs. Consequently, our model sees only about 2% (64 million / 3 billion) of the entire human genome during training.

This limited exposure to the full genome highlights the RDE model's ability to generalize from a relatively small subset of the available data. Despite seeing only a fraction of the human genome, RDE demonstrates strong performance in sequence alignment tasks across various chromosomes and even across species.

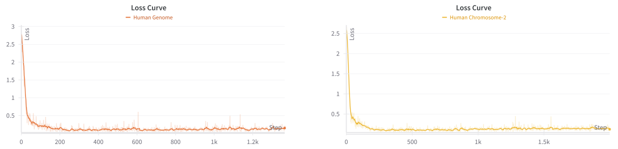

(a) Trained on the Human Genome

(b) Trained on the Human Chromosome 2

Fig. 1. RDE convergence plots. Both plots show the loss pr. step. For clarity, we smooth the loss using a moving average

B. Complexity of computing alignment

Cost of constructing a new representation

Computing the embedding $\mathcal{E}$ of a sequence of length F using RDE encoding – a typical Transformer-based attention

© The Author 2023. Published by Oxford University Press. All rights reserved. For permissions, please e-mail: journals.permissions@oup.com

architecture – has the following computation complexity:

$$\mathcal{O}(LH * (F^2 * d + d^2 * F)) \Rightarrow \mathcal{O}(F^2 * d + d^2 * F) \quad (1)$$

Where d is the embedding dimension of the model, L is the number of layers in the Transformer and H is the number of heads per layer. As d is a controllable parameter for the model, we can further simplify:

$$\mathcal{O}(F^2 * d + d^2 * F) \Rightarrow \mathcal{O}(F^2) \quad (2)$$

The F^2 complexity follows the basic implementation of attention in transformers, but recent efforts [1, 4] have developed shortcuts to reduce the cost. These have already been applied to DNA sequence modeling [8].

Vector Store Upstream

Vector store $\mathcal{D}$ is populated once (in bulk) with encoded fragment-length sequences drawn from the entire genome; *constant time complexity* C to upload $< 10M$ vectors.

Retrieval Cost

Given a new embedding, $\epsilon(G, K)$ is the cost of retrieving top-K nearest neighbors across the fragment embeddings, where G is the length of the reference genome. In modern vector databases, where several hashing techniques such as approximate K-nearest neighbors are used, ϵ scales logarithmically with G. This is indeed the key benefit of using such databases.

Fine-grained Alignment

Existing libraries/algorithms (e.g. the Smith–Waterman algorithm) can identify the alignment between a fragment sequence (of length F) and read (of length Q) in $\mathcal{O}(FQ)$.

Total Complexity

Total complexity involves (a) constructing the representation of a read; (b) querying the vector store; (c) running fine-grained alignment with respect to the K returned reference fragment sequences:

$$\mathcal{O}(F^2 + FQK + \epsilon(G, K)) \Rightarrow \mathcal{O}(F(F + QK) + \epsilon(G, K)) \\ \Rightarrow \mathcal{O}(FQK + \epsilon(G, K))$$

C. Ablation studies

Without diversity priors in top-K

In the main text, we report the performance without diversity priors used in the retrieval step: i.e. the nearest-K neighbors in the embedding space are selected from the *entire* set of fragments spanning the genome rather than uniformly sampling from each chromosome. The performance predicably falls in comparison to those reported in the main text since fewer fragments scattered unevenly across the different chromosomes are being retrieved per read.

Across read lengths

In Table 2, the performance of RDE on ESA is reported across read lengths. We observe *Zero-shot performance at longer read lengths*: The model performs better at longer read lengths (even exceeding the read length bound established during training $\mathcal{U}[150, 500]$); while evaluating for longer reads, we make sure to guarantee that the reads exist as subsequences of fragments. Improving the performance at shorter read lengths is the subject of future work.

D. Task transfer from Chromosome 2

We present two experiments that demonstrate that the model learns to solve the sequence alignment task rather than memorizing the genome on which it is trained.

Experiment setup: RDE is trained on Chromosome 2 – the longest chromosome – and recall is computed on unseen chromosomes from the human genome (3, Y) (*inter-chromosome*) and select chromosomes from chimpanzee (2A,2B) and rat (1,2) DNA (*inter-species*). Reads are generated with the following simulator configurations: $I = 10^{-2}$, $D = 10^{-2}$, $d_{SW} = 2\%$, $Q = 250$. Top-K is set to 50 / Chr., reads per setting $= 5,000$. Independent vector stores are constructed for each chromosome; representations for reference fragments (staging) and reads (testing) *are generated by the Chr. 2-trained model.* The results are reported in Tables 3, 4.

Performance: Details on convergence are in the SI Sec. A. Table 3 shows that the performance on unseen human chromosomes (3, Y) is similar to the performance reported in the main table training RDE on Chr. 2. This suggests RDE's ability to generalize sequence alignment across chromosomes with different compositions. Even distantly related species like Thermus aquaticus and Acidobacteriota show significant recall, highlighting RDE's task transferability beyond simple data memorization.

E. Potential for speed-ups in index creation and read alignment

The process of creating the ESA index involves generating embeddings for all reference fragments (Step 1), and uploading these embeddings to a vector database (Step 2). Traditional aligners like Bowtie-2 typically require 1–2 hours to construct an FM-index for the human genome on standard hardware. Currently, our RDE approach completes Step 1 in about 1 hour using a single NVIDIA A6000 GPU and takes an additional 1–2 hours to populate the Pinecone vector store. We are exploring several strategies to improve speed and efficiency without sacrificing performance:

- *Parallelized Embedding Generation:* Leveraging multi-GPU setups and high-memory GPUs could parallelize Step 1, potentially achieving a 16x speedup with an 8-GPU cluster and 2x memory capacity in each GPU.
- *High-Throughput Vector Stores:* Utilizing vector databases with faster upload and polling rates would enable simultaneous read/write of multiple fragments/embeddings, albeit at higher cloud costs. Local vector stores like FAISS are another option.
- *Model Optimization:* Techniques such as model distillation and weights quantization of the embedding model could help accelerate embedding model inference.

I	D	$Q_{PH} \in [30, 60]$ $d_{SW} < 1\%$	$d_{SW} < 2\%$	$d_{SW} < 5\%$	$Q_{PH} \in [60, 90]$ $d_{SW} < 1\%$	$d_{SW} < 2\%$	$d_{SW} < 5\%$
Top-1250 $\sim$ 50 x {Chr. 22, X, Y, M}							
0.0	0.0	98.56 ± 0.67	97.60 ± 0.85	98.56 ± 0.67	97.76 ± 0.82	97.76 ± 0.82	97.44 ± 0.88
0.01	0.01	98.8 ± 0.62	98.4 ± 0.71	98.8 ± 0.62	99.2 ± 0.52	98.4 ± 0.71	98.4 ± 0.71
Top-50 *global*							
0.0	0.0	91.6 ± 1.49	92.4 ± 1.43	93.8 ± 1.31	90.4 ± 1.58	93.8 ± 1.31	94.8 ± 1.21
0.01	0.01	89.6 ± 1.64	91.6 ± 1.49	96 ± 1.07	94.4 ± 1.25	94.4 ± 1.25	93.2 ± 1.36

Table 1. RDE performance on ESA - without diversity priors: The various parameters are described in the main text. It is evident that the addition of diversity priors results in an improvement of $\sim$ in recall. Similar to the result presented in the main text, performance improves with larger search radius in the vector store (top-K), higher quality reads (Q_{PH}) and large distance bound (d_{SW}).

I	D	$Q = 200$	$Q = 225$	$Q = 250$
$Q_{PH} \in [30, 60]$				
0	0	99 ± 0.57	98.6 ± 0.67	**99 ± 0.57**
0.01	0.01	97.4 ± 0.88	98.8 ± 0.62	**98.6 ± 0.67**
$Q_{PH} \in [60, 90]$				
0	0	98.6 ± 0.67	98.6 ± 0.67	**98.9 ± 0.59**
0.01	0.01	98.6 ± 0.67	99 ± 0.57	**98.6 ± 0.67**

Table 2. RDE recall performance on ESA across read lengths: Performance of RDE is robust across various read lengths supported by the ART simulator. Additional experiments performed using synthetically-generated "pure" reads – random subsequences of the reference genome – of length $[500, 1000]$ showed that the recall performance is high ($\geq 99\%$).

F. A setup for de-novo Genome Assembly using RDE

Consider a set of reads, $R := \{r_1, r_2, \ldots, r_C\}$ from which we would like to estimate a reference $\mathcal{R}$. The de-novo assembly task is to estimate an ordering of the reads in R, $\hat{R} := (r_i, \ldots, r_j, \ldots, r_k)$ such that a concatenation (after removing overlaps) is approximately the original reference. In typical setups, all pairs of reads would need to be compared in order to find those that overlap and construct longer chains of the assembly in an incremental fashion. This may be visualized as a complete graph K_C with C nodes (reads) and C^2 edges (constraints). To avoid the quadratic complexity, modern techniques such as *Overlap-Layout-Consensus* (OLC) or *de Bruijn Graph* (DBG) approaches ([5]) have adopted hot-start heuristics such as base overlap and shared K-mer counts to reduce the number of pairwise read comparisons.

The representation space modeled by RDE admits a comparable assembly setup. Fig. 2 suggested emergent 1D manifolds in the representation space – comprising the embeddings of the reads – $h(r_1), h(r_2), \ldots, h(r_C)$ – such that the relative *positions* of fragments along the manifold correlated to the relative *locations* of the fragments along the reference. *Therefore, a pairwise read-read comparison within a smaller radius in the embedding space would be sufficient to find reads that overlap.* And more generally, a *walk* along the 1D manifold would constitute a near-optimal assembly.

We provide an initial computational implementation of this idea: (a) The k-nearest neighbor (kNN) graph $G(R, d)$, representing the distances between close-by reads, is computed using the density-aware corrections provided by the UMAP library ([6]): graph adjacency matrices for each of the chromosomes is visualized in Fig. 2. For this figure, the several reads are ordered manually as they appear in the assembly in order to highlight the connectivity pattern; the bright off-diagonal values indicate a chain-like structure corresponding to the observed manifold; (b) A *Hamiltonian* cycle computed through the graph that has the lowest total distance corresponds to an assembly, $\hat{R}$. This involves solving the Traveling Salesman Problem (TSP) – we use the 3/2-approximate Christofides algorithm ([2]). After a walk across the reads is generated, a second pass through the walk greedily concatenates adjacent reads (r_i, r_{i+1}) by computing the alignment using the SW distance and removing duplicates and overlaps. In cases where adjacent reads do not have any alignment to one another – the walk isn't guaranteed to be perfect since the RDE model is heuristical and the TSP algorithm is approximate – the current assembly part is stowed away as a *contig* ([3]), and a new contig is instantiated with the incoming read. The fewer the contigs, the better the manifold in the representation space correlates to the ordering of the true reference.

Assembly is conducted across 4 of the shorter chromosomes in the *Thermus Aquaticus* genome. We consider (long) reads of length $Q = 500$, which have no indels, and the entire corpus of reads is ensured to have full coverage across each chromosome. Each read has an overlap of $\mathcal{U}([150, 250])$ bases with another read in the corpus of reads. QUAST ([7]) metrics are reported in Table 5.

N50 refers to the length of a contig (in bases) such that the contigs that exceed this length span 50% of the reference genome. NGA50 is a reference-aware N50 metric. The fewer contigs, large N(GA)50 scores in comparison to the large size of the chromosomes, when considered together, demonstrate that the reads are indeed ordered in a relative position-aware manner in the RDE embedding space with respect to a reference making the de-novo assembly task viable.

	Vector Store				
Read Origin	Human Chr.2	Human Chr.3	Human Chr.Y	Chimp Chr.2A	Chimp Chr.2B
Human Chr. 2	**99.5 ± 0.4**	1.5 ± 1.0	< 1	34.5 ± 6.0	43.1 ± 7.0
Human Chr. 3	1.0 ± 0.8	**99.0 ± 0.8**	< 1	1.0 ± 0.8	< 1
Human Chr. Y	1.2 ± 0.9	1.2 ± 1.1	**98.75 ± 1.00**	2.0 ± 1.9	< 1
Chimp Chr. 2A	70.0 ± 6.0	< 1	< 1	**97.0 ± 1.9**	1.5 ± 1.1
Chimp Chr. 2B	71.0 ± 6.0	< 1	< 1	1.0 ± 0.8	**95.9 ± 2.0**

Table 3. ESA Performance on Cross-Species Chromosome Alignment: RDE model trained on human chromosome-2 is evaluated for aligning reads from human chromosomes 2, 3, Y, and chimpanzee chromosomes 2A and 2B to vector stores of each these chromosomes. Values represent the percentage of reads aligned to each store (mean ± standard deviation where applicable). The results show high accuracy for intra-species alignment (99.5% for Human Chr. 2, 99.0% for Human Chr. 3, 98.75% for Human Chr. Y, 97.0% for Chimp Chr. 2A, and 95.9% for Chimp Chr. 2B). Notably, there's significant cross-species alignment between Human Chr. 2 and both Chimp Chr. 2A and 2B (34.5%, 43.1% respectively), and vice versa (70.0%, 71.0%), reflecting their evolutionary relationship. These findings indicate the RDE model's capability of aligning both positive and null reads.

**Species**	$Q_{PH} \in [30, 60]$	$Q_{PH} \in [60, 90]$
	Recall Top-K @50 ↑	
Thermus Aquaticus [All]	99.9 ± 0.01	99.9 ± 0.01
Acidobacteriota [All]	99.9 ± 0.01	99.9 ± 0.01
Rattus Norwegicus [Chr. 1]	95.98±1.07	96.48±1.01
Rattus Norwegicus [Chr. 2]	97.99±0.78	96.49±1.01
Pan Troglodytes [Chr. 2A]	98.5±0.69	95.74±1.11
Pan Troglodytes [Chr. 2B]	95.99±1.08	96.25±1.05

Table 4. Cross-Species Task Transfer with RDE on ESA: Training on Human Chr. 2, Testing on Diverse Species: RDE, trained on human Chr. 2, aligns fragments and reads from different species, including *Rattus Norwegicus, Pan Troglodytes, Acidabacteriota*, and *Thermus Aquaticus*. These species, are evaluated for read alignment recall using ART-generated reads. The findings indicate RDE's proficiency in modeling DNA sequence structure, beyond simply memorizing training data.

		Size (in kb) ↑	***No. of Contigs*** ↓	***N50 (in kb)*** ↑	***NGA50 (in kb)*** ↑
**Thermus Aquaticus**	NZ_CP010823.1	14.4	1	14.4	14.4
	NZ_CP010824.1	16.6	1	16.6	16.6
	NZ_CP010825.1	69.9	22	6.0	4.4
	NZ_CP010826.1	78.7	15	10.3	10.3

Table 5. RDE performance on de-novo assembly of the Thermus Aquaticus genome: The solution to the approximate TSP applied on the RDE read-read network generates a set of contigs that can be evaluated with respect to the original reference using QUAST [7]. For the two shorter chrosomomes, the resulting assembly was perfect; the entire reference is encapsulated by one contig. Even in the case with longer chromosomes, the number of contigs are still few and of significant length (as indicated by the high N(G)50 score) suggesting a strong correlation between the distance metric in the embedding space and the ordering of the corresponding reads in the assembly.

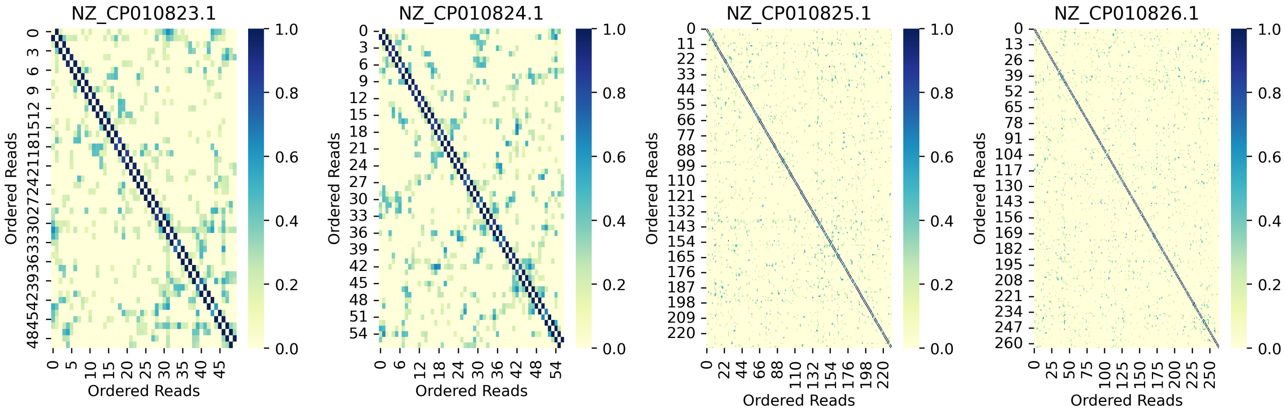

Fig. 2. Motivating the task of de-novo assembly using the distance adjacency matrix of the UMAP-generated k-nearest neighbor ($k = 10$) network of reads from Thermus Aquaticus: We assume that reads $r_1, r_2, \ldots, r_C$ belonging to a species are embedded into the RDE embedding space $h(r_1), h(r_2), \ldots, h(r_C)$ using a model trained on a known reference genome belonging to a different species. For this illustrative numerical example we ensure that reads are created with the following properties: first, no read is a substring of any other read (efficient algorithms for the case where reads can be substrings of others will be covered in our future work); second every read has a corresponding read that it overlaps with for each end, and finally, the union of the reads cover the ground-truth reference genome. Thus, for every read r_i there exist reads r_k and r_j such that the ordered triplet (r_j, r_i, r_k) constitutes a fragment of the reference. *In a perfect embedding scenario*, the closest neighbors of r_i (i.e. $k = 1$ in an k-NN search) in the embedding space should be r_j ad r_k. For this ideal case, if a network is constructed where each read (node) is connected to its two nearest neighbors, and the nodes are arranged based on the order in which they appear in the reference chromosome, one would get a bi-diagonal adjacency matrix. When ground truth is not known, the adjacency matrix will be a permuted version of a bi-diagonal matrix, but a complete assembly can still be performed by a greedy algorithm that starts with any node and pieces together the neighboring reads in the embedded space in time that grows linearly in the number of reads. Simulated reads, each of length 500 and with random overlaps of lengths drawn uniformly $\mathcal{U}([150, 250])$ with its neighboring reads at each end, are sampled from 4 of the chromosomes in Thermus Aquaticus, and are embedded into the RDE embedding space trained with a reference human genome. The sorted adjacency matrix of the UMAP ([6])-generated k-NN ($k = 10$) network is presented above for each chromosome. The dark off-diagonal distance values in the k-NN adjacency graph with $k = 10$ *show that the distance properties of actual embeddings of simulated reads from Thermus Aquaticus are almost coincident with that of the ideal case discussed above*. Moreover, just as in the ideal case, by finding an approximate solution to the Traveling Salesman Problem in the unsorted network, one can find a walk (the assembly) that is close to the original reference. The results are presented in Table 5.

References

1. Iz Beltagy, Matthew E. Peters, and Arman Cohan. Longformer: The long-document transformer. *arXiv preprint arXiv:2004.05150*, 2020.
2. Nicos Christofides. Worst-case analysis of a new heuristic for the travelling salesman problem. *Operations Research Forum*, 3, 1976.
3. Simon G Gregory. *Contig Assembly*. John Wiley & Sons, Ltd, 2005.
4. Nikita Kitaev, Lukasz Kaiser, and Anselm Levskaya. Reformer: The efficient transformer. *arXiv preprint arXiv:2001.04451*, 2020.
5. Zhenyu Li, Yanxiang Chen, Desheng Mu, et al. Comparison of the two major classes of assembly algorithms: overlap–layout–consensus and de-bruijn-graph. *Briefings in Functional Genomics*, 11(1):25–37, 12 2011.
6. Leland McInnes, John Healy, and James Melville. Umap: Uniform manifold approximation and projection for dimension reduction. *arXiv preprint arXiv:1802.03426*, 2018.
7. Alla Mikheenko, Andrey Prjibelski, Vladislav Saveliev, et al. Versatile genome assembly evaluation with QUAST-LG. *Bioinformatics*, 34(13):i142–i150, 06 2018.
8. Eric Nguyen, Michael Poli, Marjan Faizi, et al. Hyenadna: Long-range genomic sequence modeling at single nucleotide resolution. *arXiv preprint arXiv:2306.15794*, 2023.
